# Suppression of SUN2 by DNA methylation is associated with HSCs activation and hepatic fibrosis

**DOI:** 10.1038/s41419-018-1032-9

**Published:** 2018-10-03

**Authors:** Xin Chen, Wan-Xia Li, Yu Chen, Xiao-Feng Li, Hai-Di Li, Hui-Min Huang, Fang-Tian Bu, Xue-Yin Pan, Yang Yang, Cheng Huang, Xiao-Ming Meng, Jun Li

**Affiliations:** 1 School of Pharmacy, Anhui Key Laboratory of Major Autoimmune Diseases, Anhui Institute of Innovative Drugs, Anhui Medical University, Hefei, 230032 China; 2 The key laboratory of Anti-inflammatory and Immune Medicines, Anhui Medical University, Ministry of Education, Hefei, 230032 China; 3 Institute for Liver Diseases of Anhui Medical University, ILD-AMU, Anhui Medical University, Hefei, 230032 China; 4 Dept of Pharmacy, Anqing Municipal Hospital, Anqing, 246000 China

## Abstract

Hepatic myofibroblasts, activated hepatic stellate cells (HSCs), are the main cell type of extracellular matrix (ECM) deposition during hepatic fibrosis. Aberrant DNA methylation-regulated HSCs activation in liver fibrogenesis has been reported, but the functional roles and mechanisms of DNA methylation in hepatic fibrosis remain to be elucidated. In the present study, reduced representation bisulfite sequencing (RRBS) analysis of primary HSCs revealed hypermethylation patterns in hepatic fibrosis. Interestingly, we found SAD1/UNC84 domain protein-2 (SUN2) gene hypermethylation at CpG sites during liver fibrogenesis in mice with CCl_4_-induced hepatic fibrosis, which was accompanied by low expression of SUN2. In vivo overexpression of SUN2 following adeno-associated virus-9 (AAV9) administration inhibited CCl_4_-induced liver injury and reduced fibrogenesis marker expression. Consistently, in vitro experiments showed that enforced expression of SUN2 suppressed HSCs activation and exerted anti-fibrogenesis effects in TGF-β1-activated HSC-T6 cells. In addition, the signaling mechanisms related to SUN2 expression were investigated in vivo and in vitro. Methyltransferase-3b (DNMT3b) is the principal regulator of SUN2 expression. Mechanistically, inhibition of protein kinase B (AKT) phosphorylation may be a crucial pathway for SUN2-mediated HSCs activation. In conclusion, these findings provide substantial new insights into SUN2 in hepatic fibrosis.

## Introduction

Hepatic fibrosis is a key pathological feature and common cause of various chronic liver diseases^1^. Persistent liver fibrogenesis caused by the wound-healing response to liver injuries may result in liver parenchyma and vascular architecture distortions, functional impairment^2^, end-stage liver cirrhosis, or hepatocellular carcinoma^3^. The principal contributor responsible for liver fibrogenesis is excessive accumulation of extracellular matrix (ECM). More importantly, alpha-smooth muscle actin (α-SMA)-positive hepatic myofibroblasts^4^, a subset of activated hepatic stellate cells (HSCs)^5^, are the predominate regulator of the ECM during liver fibrosis^6^. Phenotypic alteration of HSCs substantially promotes fibrillar collagen deposition that ultimately induces liver fibrogenesis^7^.

HSCs activation is related to epigenetic modifications, especially DNA methylation^8^. Abnormal DNA methylation patterns of cytosines in CpG sites may trigger gene hypermethylation^9^ that impairs gene transcriptional activity^10^. Reduced representation bisulfite sequencing (RRBS) analysis, a bisulfite-based method, enriches CG-rich sites in genomes and captures the majority of promoters and other relevant regulatory regions for DNA methylation analysis^11^. Consistent with recent reports, in our present study, we identified hypermethylation patterns by RRBS in primary HSCs isolated from CCl_4_-treated mice compared with vehicle-treated mice. Interestingly, the results revealed hypermethylation of SAD1/UNC84 domain protein-2 (SUN2) during hepatic fibrosis.

SUN2, a member of the SUN domain protein family, is an integral membrane component of the inner nuclear membrane. SUN2 protein is conserved among all eukaryotes and widely expressed in various organs and tissues. Of note, SUN2 is a novel anti-cancer candidate and plays a suppressive role in central nervous system embryonal tumors^12^, breast cancer^13^ and lung cancer^14^ by inhibiting cancer cell proliferation, migration, and promoting apoptosis. Importantly, fibrogenesis is a common pathological feature of final cancer. In addition, SUN2 is required to maintain genomic stability, and deficiency of SUN2 distinctly induces DNA damage^15^, which is critically involved in hepatic fibrosis^16^. However, the roles of SUN2 in fibrotic diseases, specifically hepatic fibrosis, remain speculative. Considering the evidence indicating SUN2 hypermethylation in hepatic fibrosis mice, we hypothesized that silencing of the SUN2 gene by DNA hypermethylation may be associated with HSCs activation and liver fibrogenesis. In this study, we investigated the functions and molecular mechanisms of SUN2 in hepatic fibrosis.

## Results

### Verification of the CCl_4_-induced hepatic fibrosis model in mice

**Fig. 1:**
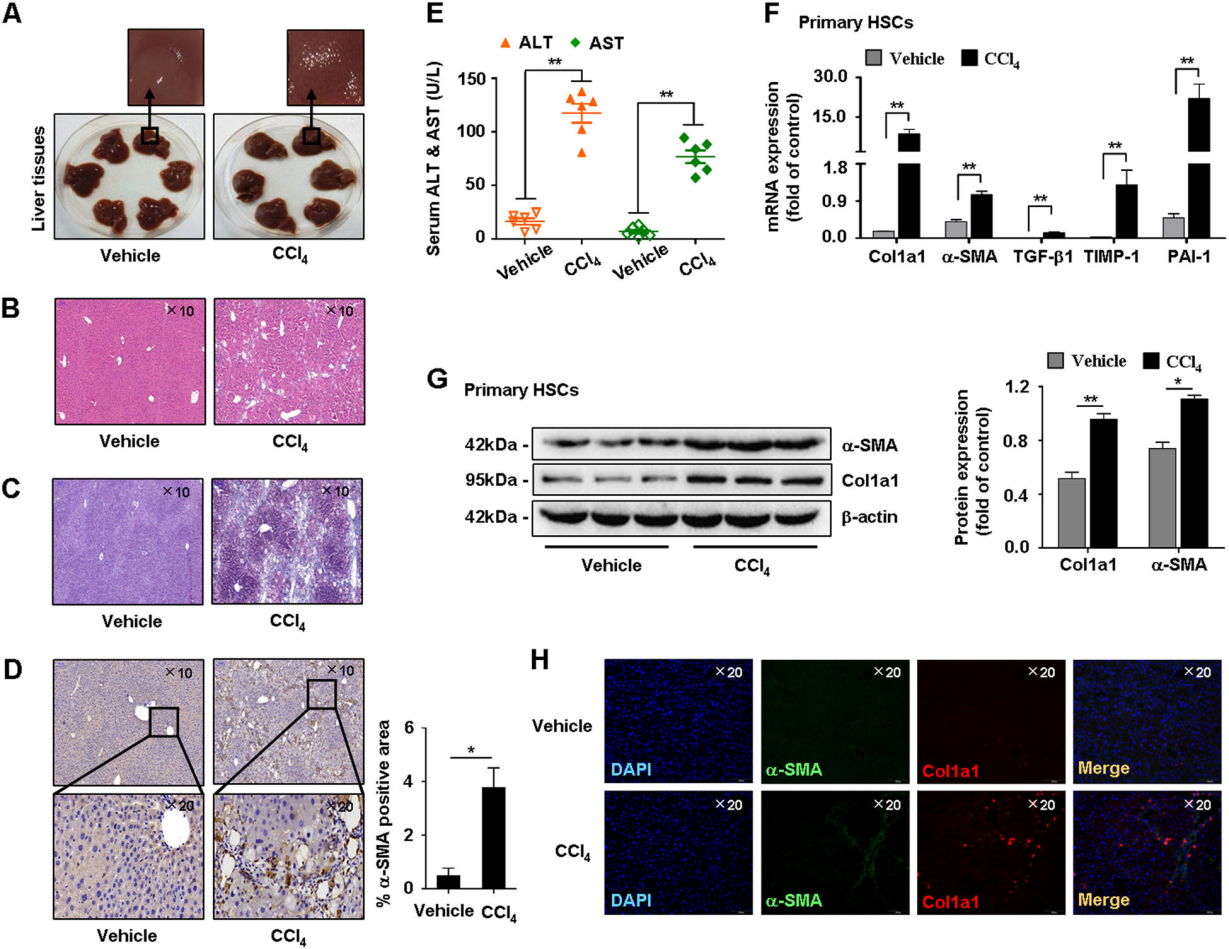
Characterizations of CCl_4_-induced hepatic fibrosis mice model. **a** Representative images of fresh livers from CCl_4_-treated mice and vehicle. **b, c** Pathology observation of H&E staining and Masson staining of mice liver tissues, representative views were presented, scale bar, 100 μM. **d** Immunohistochemistry showed α-SMA was higher expressed in CCl_4_-treated mice compared with vehicle. Representative views were presented, scale bar, 100 μM (up) and 50 μM (down). **e** Test of serum ALT and AST levels. **f** Quantitative real-time PCR detected the mRNA levels of α-SMA, Col1α1, TGF-β1, TIMP-1, and PAI-1. **g** Western blot analysis showed protein expression of α-SMA and Col1α1. **h** Immunofluorescence staining showed higher fluorescence intensity of α-SMA and Col1α1 in CCl_4_-treated mice compared with vehicle. Representative views were presented, scale bar, 100 μM. Graphs showed quantification of western blotting for α-SMA and Col1α1. Bars represent mean ± SEM for 6 mice. *p < 0.05; **p < 0.01 versus vehicle

First, pathological characteristics of the mice were investigated. Histologically, hematoxylin & eosin (H&E) and Masson staining showed liver injury and elevated collagen deposition in CCl_4_-treated mice compared with vehicle (Figs. 1b, c). Immunostaining of α-SMA and serum levels of ALT and AST were increased in CCl_4_-treated mice (Figs. 1d, e). Additionally, mRNA levels of fibrogenic factors α-SMA, type I collagen (Col1α1), transforming growth factor-β1 (TGF-β1), tissue inhibitor of metallopeptidase-1 (TIMP-1), and plasminogen activator inhibitor-1 (PAI-1) were significantly upregulated in primary HSCs isolated from CCl_4_-treated mice (Fig. 1f). Immunoblotting showed that expression of α-SMA and Col1α1 was increased in CCl_4_-treated mice (Fig. 1g). Furthermore, immunofluorescence (IF) analysis showed that α-SMA and Col1α1 were consistently increased in CCl_4_-treated mice (Fig. 1h). These results indicated successful establishment of the CCl_4_-induced hepatic fibrosis model in mice.

### **RRBS analysis of DNA methylation patterns in primary HSCs from vehicle-treated and** hepatic fibrosis **model mice**

**Fig. 2:**
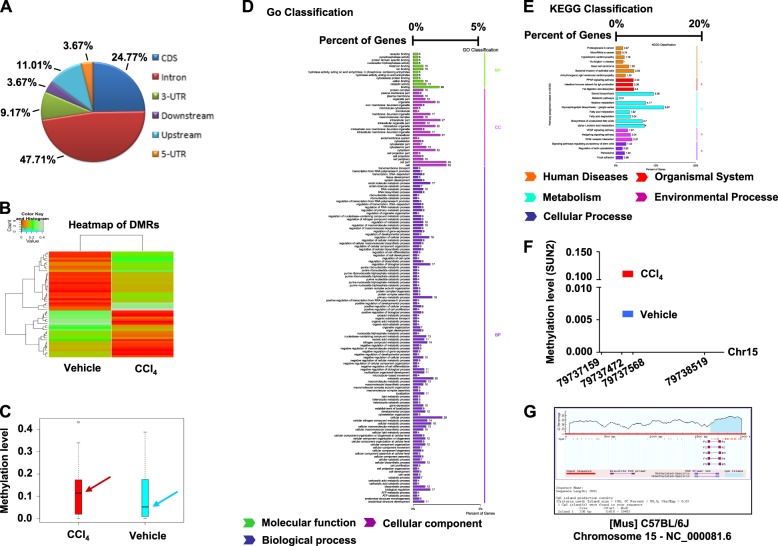
DNA methylation patterns were identified by RRBS analysis in primary HSCs. **a** Distribution of differentially methylated regions (DMRs) located at diverse genomic regions, CDS (24.77%), intron (47.71%), 3‵-UTR (9.17%), downstream (3.67%), upstream (11.01%) and 5‵-UTR (3.67%), respectively. **b** Heatmap of DMRs in two groups. **c** Overall methylation level comparison of DMRs, box plot showed hypermethylation patterns of hepatic fibrosis mice compared with vehicle. **d, e** Enrichments of GO analysis and KEGG analysis for differentially expressed genes in DMRs. **f** Methylation level of SUN2, SUN2 gene present hypermethylated in hepatic fibrosis mice compared to vehicle. **g** Prediction of SUN2 methylation CpG sites in C57BL/6 J mouse

To assess potential regulation of DNA methylation in progression of hepatic fibrosis, RRBS analysis of DNA methylation sites was performed in primary HSCs isolated from vehicle- and CCl_4_-treated mice. Figures 2a, b showed the distribution of differentially methylated regions (DMRs) at various genomic regions and a heatmap of DMRs in the two groups. Primary HSCs from hepatic fibrosis mice exhibited overall hypermethylation patterns compared with vehicle-treated mice (Fig. 2c). We next detected DMRs in CpG island (CGI) sites and found 38 abnormally methylated genes in hepatic fibrosis model mice (Supplementary Table 1). In addition, GO and pathway analyses of hypermethylated genes were performed (Figs. 2d, e). Furthermore, as promising biomarkers of hepatic fibrosis, 33 CpG sites were hypermethylated in the SUN2 gene of DNA samples from hepatic fibrosis model mice compared with vehicle-treated mice (Fig. 2f and Supplementary Table 1). Prediction of methylated CpG sites of SUN2 in the C57BL/6 J mouse is shown in Fig. 2g. Considering hypermethylation of SUN2 was found in hepatic fibrosis mice, we next investigated the potential functions and relevant mechanisms of SUN2 in hepatic fibrosis.

### Effects of downregulation and overexpression of SUN2 in hepatic fibrosis mice on liver fibrogenesis

**Fig. 3:**
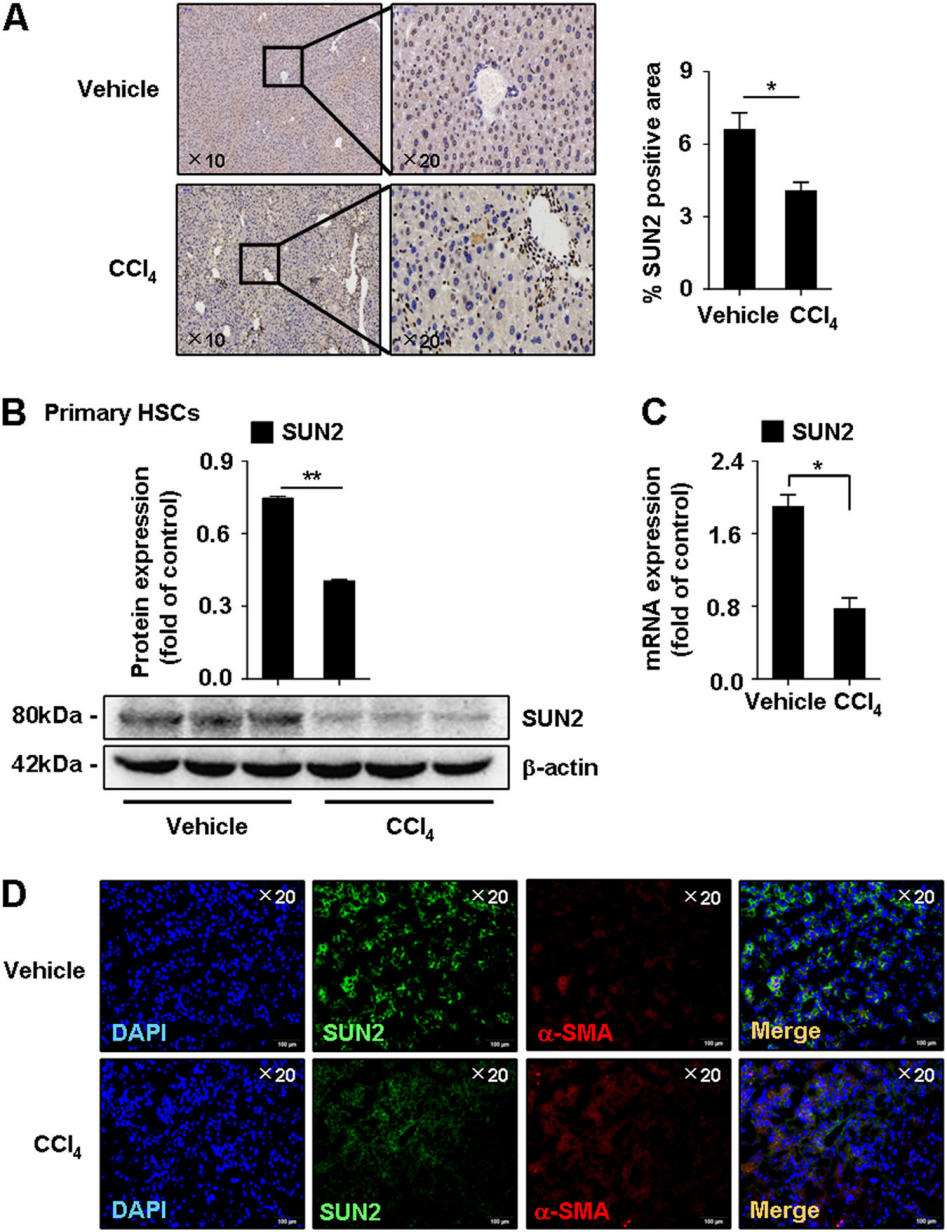
**Down-regulation of SUN2 in hepatic fibrosis mice**. **a** Immunohistochemistry of SUN2 in mice liver tissue. Representative views were presented, scale bar, 100 μM (left) and 50 μM (right). **b, c** Lower mRNA level and protein expression of SUN2 in primary HSCs from hepatic fibrosis mice compared with vehicle. **d** Double-immunofluorescence showed representative colocalization of SUN2 with α-SMA in liver tissue. Representative views were presented, scale bar, 100 μM. Graphs showed quantification of western blotting for SUN2. Data represent results from groups of 6 mice and each bar resents mean ± SEM. *p < 0.05; **p < 0.01 versus vehicle

Next, expression of SUN2 was determined in mouse liver fibrogenesis. Lower SUN2 staining by immunohistochemistry was observed in hepatic fibrosis mice compared with vehicle-treated mice (Fig. 3a). In addition, mRNA and protein expression of SUN2 was significantly downregulated in primary HSCs from hepatic fibrosis mice (Figs. 3b, c). Moreover, double IF analysis showed colocalization of SUN2 (green) with myofibroblast marker α-SMA (red) immunoreactivity in mouse liver tissues (Fig. 3d).

**Fig. 4:**
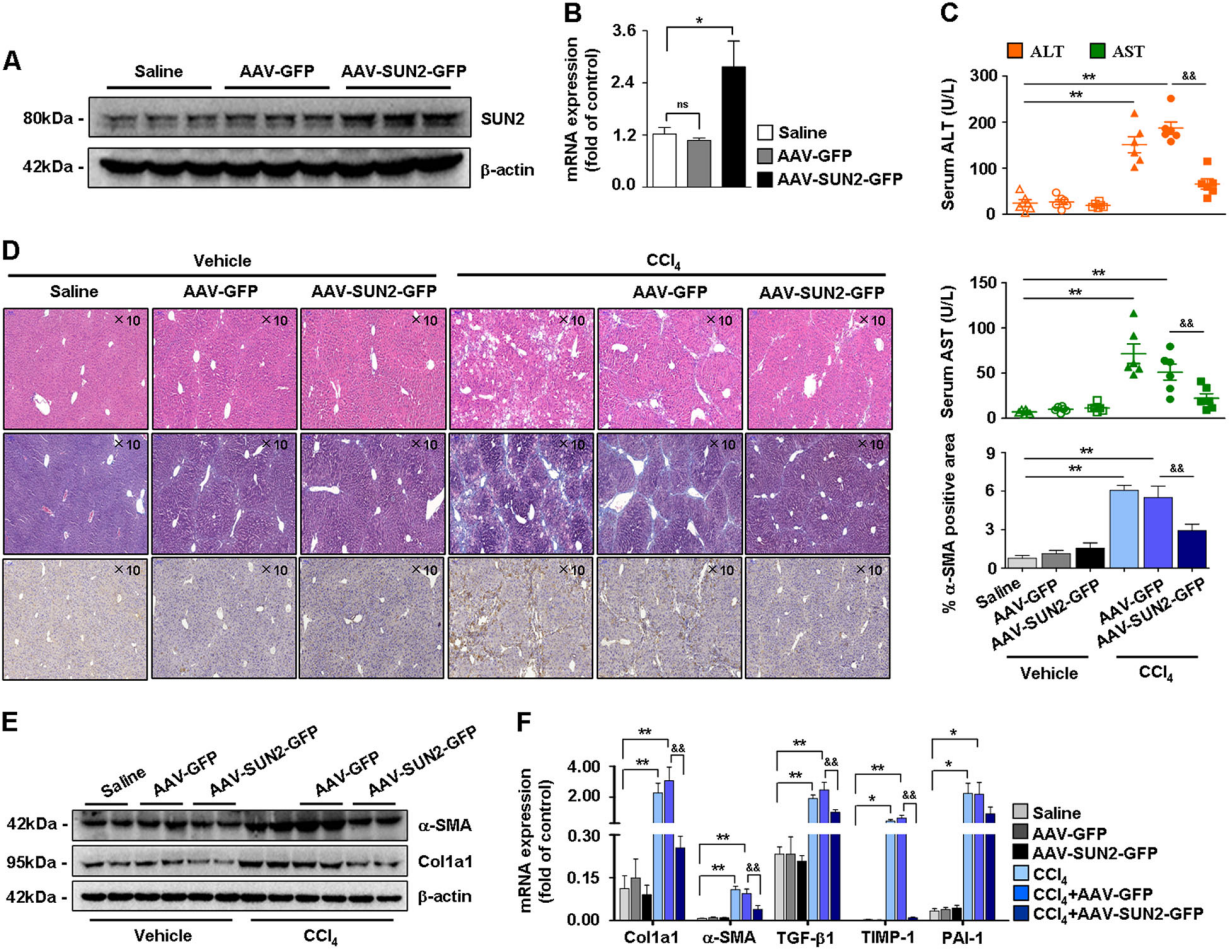
Effects of SUN2 overexpression on hepatic fibrosis mice. **a, b** Efficacy of AAV-SUN2-GFP delivery in mice, western blot analysis and quantitative real-time PCR showed expression of SUN2 enhanced in mice with AAV-SUN2-GFP administration. **c** Test of serum ALT and AST levels. **d** Pathology observation of H&E staining and Masson staining in CCl_4_-treated mice following AAV-SUN2-GFP delivery, and immunohistochemistry staining of α-SMA. Representative views were presented, scale bar, 100 μM. **e, f** Compared with AAV-GFP (empty vector), both mRNA levels and protein expression of fibrogenic genes were attenuated in CCl_4_-treated mice with AAV-SUN2-GFP administration. Graphs showed quantification of western blotting for SUN2, α-SMA, Col1α1. Bars represent mean ± SEM for six mice. *p < 0.05; **p < 0.01 versus Saline; ^**&**^*p* < 0.05; ^**&&**^*p* < 0.01 versus CCl_4_ + AAV-GFP

Based on these observations, SUN2 was overexpressed in mice by systemic administration of an adeno-associated virus-9 (AAV9) vector. The effect of AAV-SUN2-GFP delivery on SUN2 expression was assessed (Figs. 4a, b). Histologically, liver injury was decreased and collagen deposition was attenuated in hepatic fibrosis mice following AAV-SUN2-GFP administration (Figs. 4c, d). In addition, immunohistochemical analysis showed markedly reduced α-SMA staining in liver tissues after AAV-SUN2-GFP delivery (Fig. 4d). Moreover, mRNA levels of fibrogenic genes (α-SMA, Col1α1, TGF-β1, TIMP-1, and PAI-1) and protein expression of α-SMA and Col1α1 were inhibited in mice following AAV-SUN2-GFP delivery (Figs. 4e, f). Taken together, these results revealed a remarkably lower expression level of SUN2 during liver fibrogenesis, and that AAV-mediated SUN2 overexpression suppressed liver injury and myofibroblast marker expression in hepatic fibrosis mice.

### **Lower expression of SUN2 in TGF-β1-activated HSC-T6 and LX-2 cellsin vitro**

**Fig. 5:**
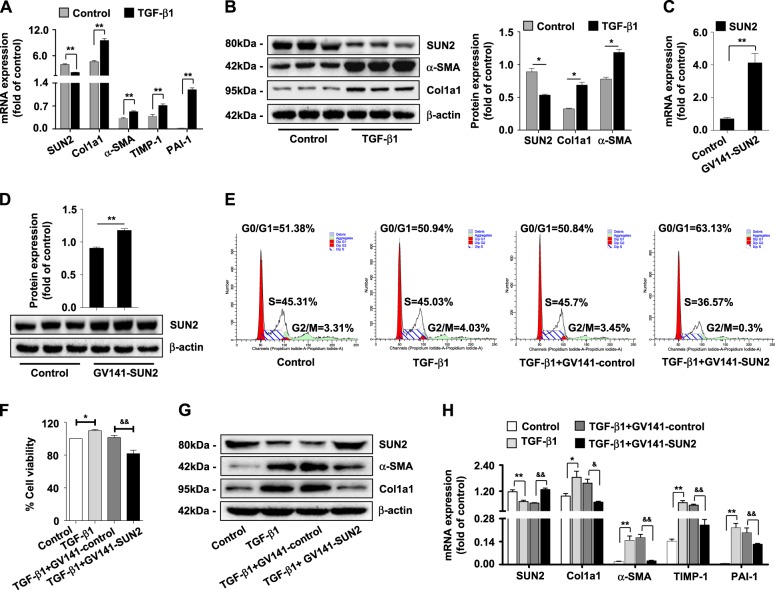
Lower expression of SUN2 in TGF-β1-activated HSC-T6 cells in vitro. **a** Quantitative real-time PCR and **(b)** western blot analysis showed that expression of fibrogenic factors elevated but SUN2 decreased in TGF-β1 (10 ng/ml)-treated HSC-T6 cells compared with control. **c, d** Characterizations of SUN2 overexpression in HSC-T6 cells transfect with GV141-SUN2 plasmid. **e** Flow cytometry identified an increased numbers of cells in G1 following transfect with GV141-SUN2 plasmid compared with GV141-control. **f** MTT assay showed that enforcing expression of SUN2 significantly inhibited viability of HSC-T6 cells. **g, h** Enforcing expression of SUN2 decreased mRNA level of α-SMA, Col1α1, TGF-β1, TIMP-1, and PAI-1 and protein expression of α-SMA and Col1α1 in TGF-β1-activated HSC-T6 cells with GV141-SUN2 plasmid transfection. Graphs showed quantification of western blotting for SUN2, α-SMA and Col1α1. Bars represent mean ± SEM for 3 independent experiments in vitro. *p < 0.05; **p < 0.01 versus Control; ^**&**^*p* < 0.05; ^**&&**^*p* < 0.01 versus TGF-β1 + GV141-control

HSC-T6 and LX-2 cells were activated with 10 and 15 ng/ml TGF-β1, respectively. Expression of fibrogenic genes (α-SMA, Col1α1, TGF-β1, TIMP-1, and PAI-1) was elevated significantly, while SUN2 expression was decreased in TGF-β1-activated HSC-T6 cells compared with the control (Figs. 5a, b). We also found lower expression of SUN2 in TGF-β1-activated LX-2 cells (Supplementary Figure S2E and S2F). These results were consistent with our earlier findings showing that SUN2 expression was decreased during hepatic fibrosis.

### SUN2 negatively regulates activation and proliferation of HSC-T6 cells

To ascertain potential functions of SUN2 in HSC-T6 cells, we performed GV141-SUN2 plasmid transfection to enforce SUN2 expression (Figs. 5c, d). Flow cytometric analysis of transfected cell populations revealed fewer cells in S phase and increased numbers of cells in G1 phase following transfection with the GV141-SUN2 plasmid compared with cells transfected with GV141-control, suggesting that enforced expression of SUN2 induced G1 arrest in HSC-T6 cells (Fig. 5e). Additionally, MTT assays showed a distinct decrease in the viability of TGF-β1-activated HSC-T6 cells transfected with GV141-SUN2 plasmid (Fig. 5f). mRNA levels of fibrogenic factors (α-SMA, Col1α1, TGF-β1, TIMP-1, and PAI-1) and protein expression of α-SMA and Col1α1 were also dramatically decreased in TGF-β1-activated HSC-T6 cells after GV141-SUN2 plasmid transfection (Figs. 5g, h).

Conversely, knockdown of SUN2 by SUN2-RNAi transfection increased HSC-T6 cell numbers in S and G2 phases compared with Scrambled-RNAi transfection in vitro (Supplementary Figure S3A and S3C). Viability of HSC-T6 cells was elevated (Supplementary Figure S3B), and expression of α-SMA, Col1α1, and TIMP-1 was elevated substantially (Supplementary Figure S3D and S3E) following SUN2 knockdown. Furtherover, enforcing or blocking expression of SUN2 exerted no significant effects on apoptosis of activated HSC-T6 cells (Supplementary Figure S4A). Collectively, these results suggest that forced expression of SUN2 suppresses TGF-β1-activated HSC-T6 cell activation and the severity of hepatic .fibrosis.

### DNA methyltransferases mediate expression of SUN2 in hepatic fibrosis mice

**Fig. 6:**
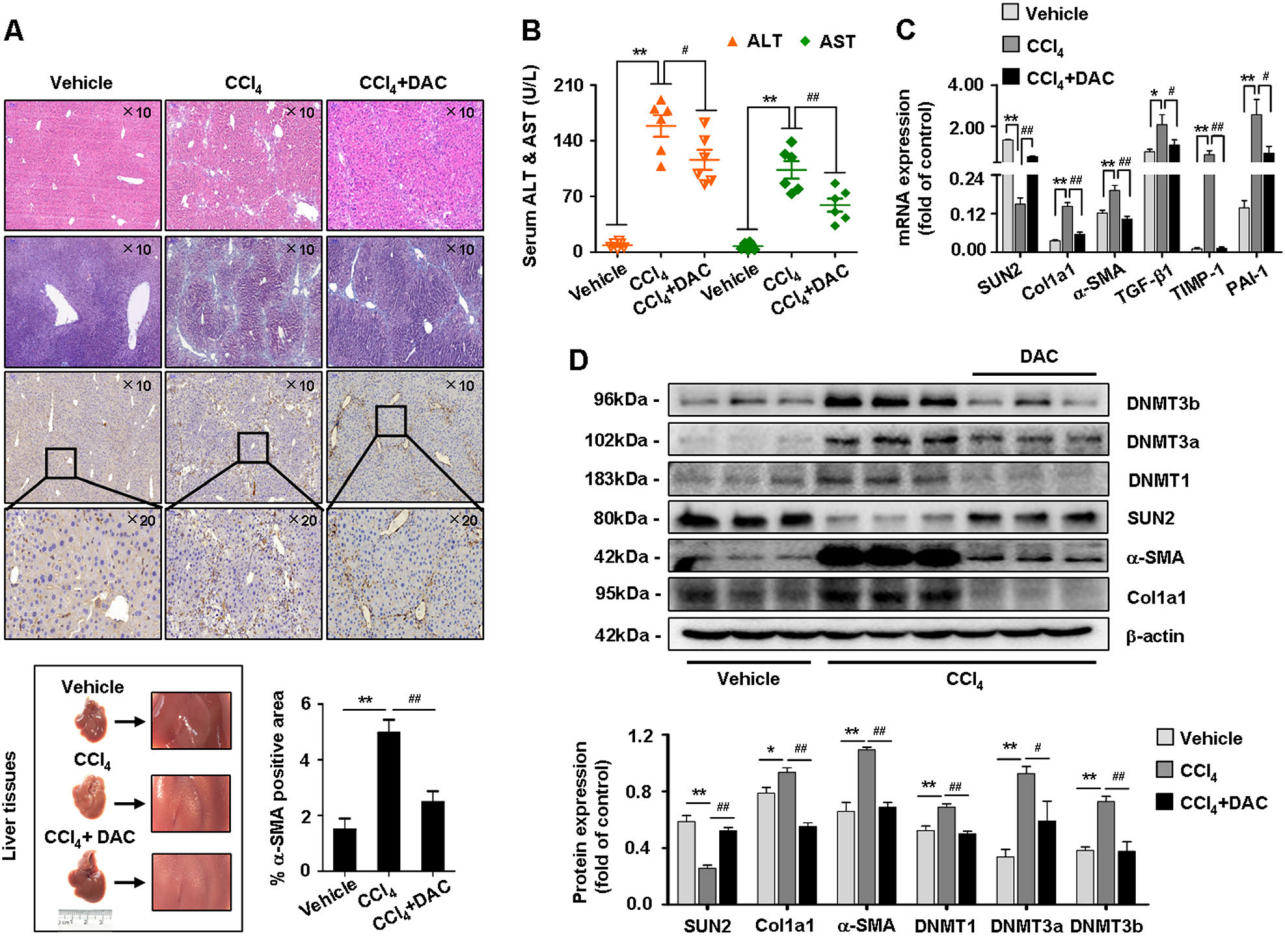
**Suppression of DNA methyltransferases ameliorated mice hepatic fibrosis**. **a** Representative images of fresh livers, H&E staining, Masson staining and IHC analysis for α-SMA were performed in vehicle, hepatic fibrosis mice and hepatic fibrosis mice with DAC treatment, scale bar, 100 μM (up) and 50 μM (down). **b** Test of serum ALT and AST levels. **c** Hepatic fibrosis mice treated with DAC exhibited mRNA levels diminution of α-SMA, Col1α1, TGF-β1, TIMP-1, and PAI-1. **d** Western blot showed expression of DNMT1, DNMT3a, and DNMT3b were decreased but SUN2 was restored in primary HSCs from CCl_4_-treated mice with DAC compared to hepatic fibrosis mice. Data represent results from groups of six mice and each bar resents mean ± SEM. *p < 0.05; **p < 0.01 versus vehicle; ^#^*p* < 0.05; ^##^*p* < 0.01 versus CCl_4_

We next assessed potential molecular mechanisms underlying the low expression of SUN2 during hepatic fibrosis. Evidence has confirmed that DNMTs contribute to DNA hypermethylation^17^. Therefore, in our study, DAC (1 mg/kg, biweekly for 4 weeks)^18^, an inhibitor of DNMTs, was intraperitoneally injected into mice with CCl_4_-induced hepatic fibrosis. H&E and Masson staining showed reduced liver injury and fibrosis in hepatic fibrosis mice treated with DAC compared with CCl_4_ only-treated mice (Fig. 6a). In addition, immunostaining of α-SMA and serum levels of ALT and AST were decreased in DAC-treated mice (Figs. 6a, b). Importantly, expression of SUN2 was restored following inhibition of DNMT1, DNMT3a, and DNMT3b in vivo (Figs. 6c, d). In parallel, DNMTs blockade led to decreases of fibrogenic factor expression (Fig. 6c, d). Together of the results suggested that the effects of DNA methyltransferases on gene hypermethylation may one of the potentially mechanism responsible for low expression of SUN2 in mouse liver fibrogenesis.

### DNMT3b negatively regulates SUN2 in HSC-T6 cells

**Fig. 7:**
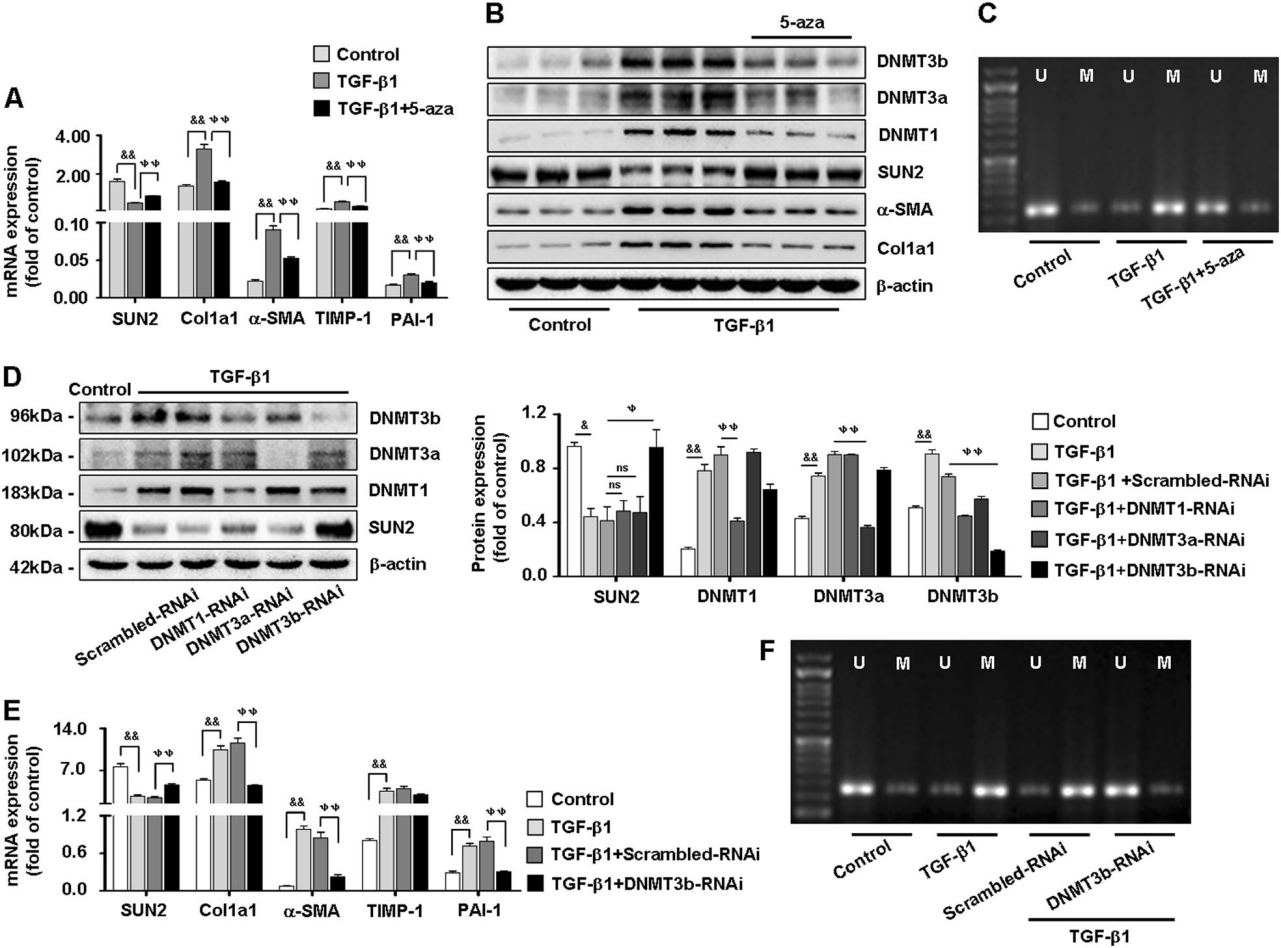
DNMT3b negatively regulates to SUN2 in HSC-T6 cells in vitro. **a, b** Both quantitative real-time PCR and western blot showed that compared with TGF-β1-activated HSC-T6 cells, expression of DNMT1, DNMT3a, and DNMT3b were decreased but SUN2 restored following 5-aza (1 μM) treatment. **c** MSP analysis detected CpG methylation level of SUN2 reversed in TGF-β1-activated HSC-T6 cells with 5-aza treatment. **d** Effects of DNMT1-RNAi, DNMT3a-RNAi, and DNMT3b-RNAi on SUN2 protein expression. **e** Silencing of DNMT3b distinctly restored mRNA level of SUN2 and attenuated α-SMA, Col1α1, TGF-β1, and PAI-1 expression. **f** Effects of DNMT3b-RNAi on methylation level of SUN2. Graphs showed quantification of western blotting for SUN2, α-SMA and Col1α1. Bars represent mean ± SEM for 3 independent experiments, in vitro. ^**&**^*p* < 0.05; ^**&&**^*p* < 0.01 versus Control; ^**ψ**^*p* < 0.05; ^**ψψ**^*p* < 0.01 versus TGF-β1 + Scrambled-RNAi

Furthermore, consistent with our earlier results, expression of DNMT1, DNMT3a, and DNMT3b was elevated in TGF-β1-activated HSC-T6 cells. SUN2 expression was restored in activated HSC-T6 cells treated with 5-azacytidine (5-aza; 1 μM, 48 h)^19^ (Figs. 7a, b). In addition, methylation-special PCR (MSP) showed that hypermethylation of SUN2 was reversed following treatment of activated HSC-T6 cells with 5-aza (Fig. 7c).

Next, DNMT1, DNMT3a, and DNMT3b were effectively knocked down (Fig. 7d). Remarkably, silencing of DNMT3b distinctly restored SUN2 expression (Fig. 7d) and attenuated expression of fibrogenic factors (α-SMA, Col1α1, TGF-β1, and PAI-1) (Fig. 7e). Additionally, DNMT3b-related methylation of the SUN2 gene was analyzed by MSP (Fig. 7f). Collectively, expression of SUN2 was associated with DNA hypermethylation, which verified the results of RRBS, and DNMT3b may be crucial for regulation of SUN2.

### Interacting with AKT signaling is one of the mechanisms by which SUN2 suppresses HSCs activation

**Fig. 8:**
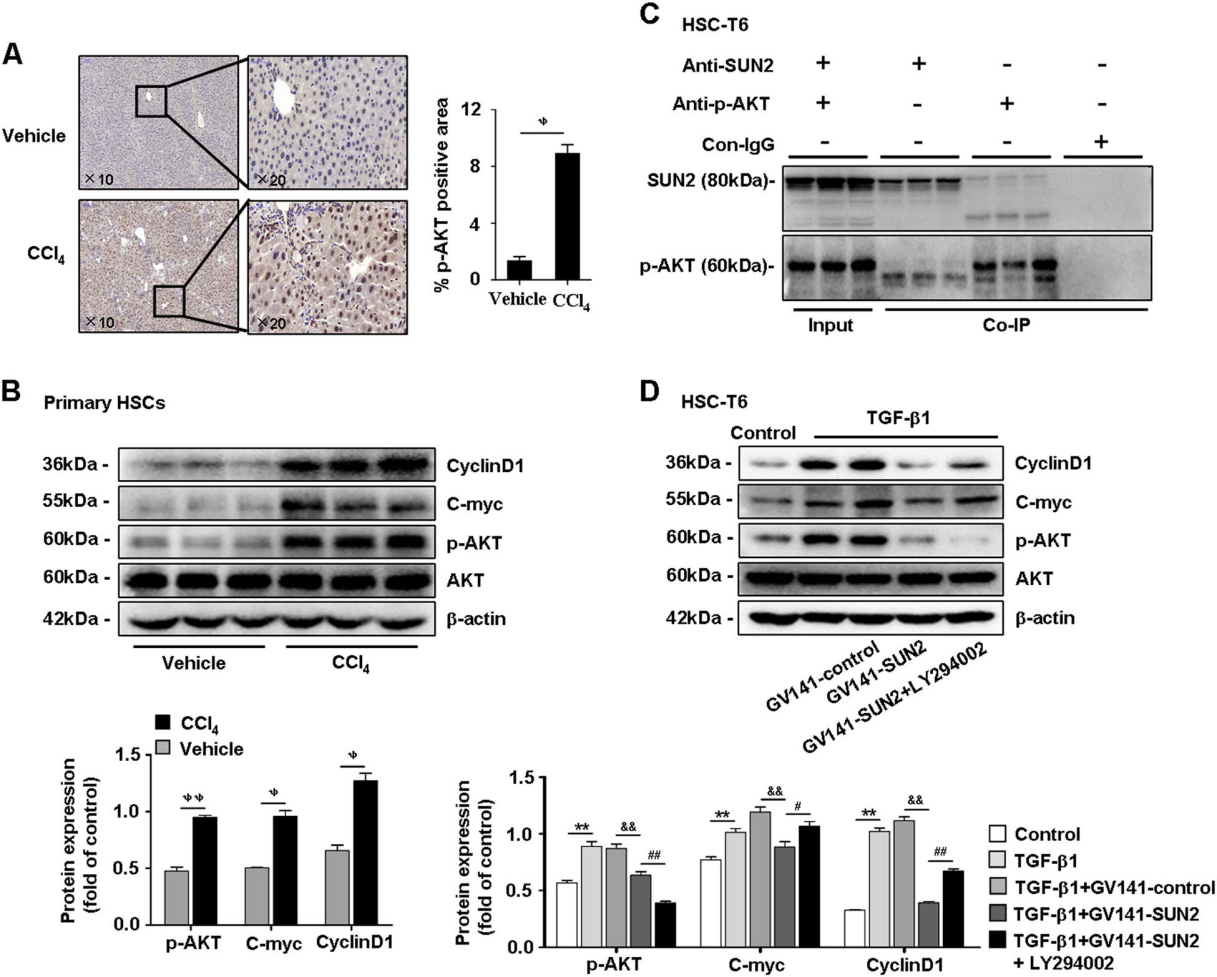
**SUN2 interacts with AKT signaling**. **a** Immunohistochemistry showed that p-AKT was highly expressed in hepatic fibrosis mice compared with vehicle. Representative views were presented, scale bar, 100 μM (left) and 50 μM (right). **b** Western blotting showed expression of p-AKT, C-myc, and CyclinD1 substantially elevated in primary HSCs from hepatic fibrosis mice in vivo. **c** Co-IP assay detected an interaction of SUN2 with p-AKT. **d** Effects of LY294002 (25 μM) on GV141-SUN2 plasmid transfected HSC-T6 cells, expression of C-myc and CyclinD1 restored following PI3K/AKT was blocked in vitro. Graphs showed quantification of western blotting for SUN2, p-AKT, C-myc, and CyclinD1. Bars represent mean ± SEM for six mice, in vivo; and three independent experiments, in vitro. ^**ψ**^*p* < 0.05; ^**ψψ**^*p* < 0.01 versus vehicle; *p < 0.05; **p < 0.01 versus Control; ^**&&**^*p* < 0.01 versus TGF-β1 + GV141-control; ^#^*p* < 0.05; ^##^*p* < 0.01 versus TGF-β1 + GV141-SUN2

Activation of the phosphatidylinositol 3-kinase/protein kinase B (PI3K)/AKT pathway promotes phenotypic alteration of HSCs and hepatic fibrosis^20^. KEGG pathway enrichment analysis of hypermethylated genes revealed that PI3K/AKT signaling was involved in mouse hepatic fibrosis (Supplementary Table 2). Consistently, immunostaining of p-AKT was increased in mice with CCl_4_-induced hepatic fibrosis (Fig. 8a). Paralleling expression of p-AKT, C-myc and CyclinD1 were increased in primary HSCs from hepatic fibrosis mice and activated HSC-T6 cells in vivo and in vitro (Fig. 8b and Supplementary Figure S4C). Furthermore, co-immunoprecipitation revealed an interaction between SUN2 and p-AKT (Fig. 8c). A previous study confirmed that activation of AKT signaling is mediated by C-myc, and cyclinD1 that are related to the cell cycle^21^. Western blotting showed that expression of p-AKT, C-myc, and CyclinD1 was reduced in TGF-β1-activated HSC-T6 cells transfected with the GV141-SUN2 plasmid compared with GV141-control (Fig. 8d). p-AKT was distinctly suppressed in HSC-T6 cells exposed to LY294002 (Fig. 8d), an inhibitor of PI3K/AKT^22^. Interestingly, expression of C-myc and CyclinD1 was restored following blockade of PI3K/AKT in HSC-T6 cells with enforced SUN2 expression. Taken together, these results suggested that phosphorylation and activation of AKT may be one of responsible pathway for SUN2-regulated amelioration of hepatic fibrosis features.

## Discussion

Quiescent HSCs phenotypically transdifferentiate into hepatic myofibroblasts^23^, which are highly proliferative cells that secrete ECM, contributing to promotion of hepatic fibrosis^24^. Researchers have persuasively demonstrated that DNA hypermethylation is a crucial step for transdifferentiation of HSCs^25^. Here, to clarify methylation patterns and provide new insights into potential biomarkers of hepatic fibrosis, we performed RRBS screening of primary HSCs isolated from mice with CCl_4_-induced hepatic fibrosis and vehicle-treated mice. Our results supported that DNA hypermethylation occurs in liver fibrogenesis. Notably, we found that SUN2 had aberrant hypermethylation in DNA samples from hepatic fibrosis mice compared with vehicle. Studies suggest that SUN2 participates in central nervous system embryonal tumors^12^, breast cancer^13^, and lung cancer^14^ by regulating biological processes in cancer^26^. In addition, recent reports show that SUN2 plays prominent roles in resistance to excessive DNA damage, one of the hallmarks of liver fibrogenesis. However, the functions and relevant mechanisms of SUN2 in hepatic fibrosis had not been investigated.

Our study provides initial evidence of SUN2 downregulation in HSCs from hepatic fibrosis mice compared with vehicle-treated mice. Functionally, significant effects of SUN2 overexpression by alleviation of liver injury and suppression of fibrosis were confirmed in hepatic fibrosis mice following AAV-SUN2-GFP administration. Aberrant alteration of SUN2 expression led us to explore potential regulation of SUN2 in HSCs activation. Expression of myofibroblast markers was inhibited in TGF-β1-activated HSC-T6 cells with enforced SUN2 expression. Moreover, overexpression of SUN2 induced G1 arrest in the cell cycle and reduced cell viability with a negligible direct influence on apoptosis of HSC-T6 cells. Taken together, our study indicated regulation of SUN2 in HSCs activation and amelioration of the pathogenesis of hepatic fibrosis in mice.

Previous reports demonstrated the relevance of PI3K/AKT signaling activation in HSCs phenotypic alteration and hepatic fibrosis^27^. Interestingly, an interaction was found between SUN2 and AKT signaling. Consistent with recent reports, phosphorylation of AKT was dramatically reduced following SUN2 overexpression in HSCs. These results revealed new evidence that phosphorylation and activation of AKT may be crucial for SUN2-regulated HSCs activation.

In conclusion, we revealed for the first time that a potential role of SUN2 in fibrotic diseases, particularly hepatic fibrosis. We found low expression of SUN2 in hepatic fibrosis mice. Notably, enhancing SUN2 expression exerted anti-fibrogenesis effects and attenuated HSCs activation, the principal contributor to hepatic fibrosis. Collectively, these results highlight SUN2 as an anti-fibrogenesis factor that may be a promising therapeutic biomarker for treatment of hepatic fibrosis. Furthermore, persistent fibrogenesis is a crucial driving force of liver cirrhosis and hepatocellular carcinoma, ultimately. Notwithstanding, hepatic fibrosis is a reversible process following withdrawal of etiological damage. Our future studies will assess the role of SUN2 in reversing progression of hepatic fibrosis.

## Materials and methods

### Mouse model of hepatic fibrosis

Eight to ten weeks old littermate male C57BL/6 J mice were used in this study. To induce hepatic fibrosis mice, CCl_4_ was dissolved in olive oil at 10% (*v/v*) and injected intraperitoneally at a dose of 0.001 ml/g, biweekly. Vehicle mice received the same volume of olive oil only. Mice with liver-specific AAV9-GFP administration were generated by tail vein injections. Groups of 8-10 mice were sacrificed at day 4 weeks after CCl_4_ treatment and liver tissues were collected. The experimental procedures were approved by Animal Experimentation Ethics Committee of Anhui Medical University.

### Primary HSCs isolation

Primary HSCs were isolated from mice as previously described^28^. Liver of mice was digested with Collagenase IV (Sigma-Aldrich, St. Louis, USA) and Pronase E (Sigma-Aldrich, St. Louis, USA) dissolved in PB buffer. Suspension of dispersed cells was layered by gradient centrifugation in Nycodenz (Axis-Shield Diagnostics, Oslo, Norway) according to manufacture protocols.

### Reduced representation bisulfite sequencing (RRBS)

Genomic DNA was extracted from primary HSCs using DNA extraction kit (Genaray, Co. Shanghai, China). RRBS was performed as previously described^29^. Length distribution of RRBS libraries were checked by Bioanalyzer (Agilent Technologies). Sequencing reads were converted and loaded on Illumina HiSeq 2000 platform. DMRs analysis was performed by swDMR software. Pipelines experimental procedures and bioinformatics analysis were shown in Supplementary File 1.

### **RNA extraction and quantitative real-time PCR**

Total RNA was isolated from primary HSCs and cultured cells using Trizol reagent^30^ (Invitrogen, Carlsbad, CA) according to the manufacturer’s protocol. Quantitative real-time PCR analysis of SUN2, α-SMA, Col1α1, TGF-β1, TIMP-1 and PAI-1 were performed as previously described^31^. The primers used in this study were list in Supplementary Table 3 A. The ratio for the mRNA interested was normalized with GAPDH.

### Western blot

Protein from primary HSCs and cultured cells was extracted using RIPA lysis buffer^32^. Concentration of protein was quantifed using NanoDrop 2000 (Thermo, California, USA). Antibodies used in this study included primary antibodies specific for SUN2, DNMT1, DNMT3a and DNMT3b (Abcam, Cambridge, UK); total AKT, phosphorylated-AKT (Cell Signaling, Danvers, MA); α-SMA, Col1α1 (Bioss, Beijing, China), β-actin (Bioworld, Minnesota, USA). Signal intensities of each western blot were quantified by using the Image J software (NIH, Bethesda, MD, USA).

### Methylation-special PCR (MSP)

DNA samples treated with Wizard^®^ DNA Clean-Up System (Promega, Co. Madison, USA) according to the manufacturer’s protocols. Conversion of unmethylated cytosine to uracil using Methylamp™ DNA Modification Kit (Epigentek, Inc. USA) in purified DNA samples. Primers of methylated and unmethylated SUN2 were listed in Supplementary Table 3B.

### Histology and immunohistochemistry

Paraformaldehyde-fixed, paraffinembedded liver tissues were sectioned (4 μm) for H&E staining and Masson staining as described previously^33^. Sections were examined using an automatic digital slide scanner (Model: pannoramic MIDI, 3DHISTECH, Hungary). IHC staining of SUN2, α-SMA and phosphorylated-AKT were performed using a microwave-based antigen retrieval technique^34^.

### Statistical analysis

Data collected from this study were expressed as mean ± SEM and analyzed using one-way analysis of variance (ANOVA), followed by Newman–Keuls post-hoc test (Prism 5.0 GraphPad Software, Inc, San Diego, CA, USA).

## Electronic supplementary material

Figure legends

Figure legends

Supplementary Figure 1

Supplementary Figure 2

Supplementary Figure 3

Supplementary Figure 4

Supplementary Figure legends

Supplementary Figure legends

Supplementary Table 1

Supplementary Table 2
