# Supplementary figures and images for: Suppression of SUN2 by DNA methylation is associated with HSCs activation and hepatic fibrosis
Source: Cell Death Dis. 2018 Oct 3;9(10):1021. doi: 10.1038/s41419-018-1032-9 (PMC6170444; doi:10.1038/s41419-018-1032-9)

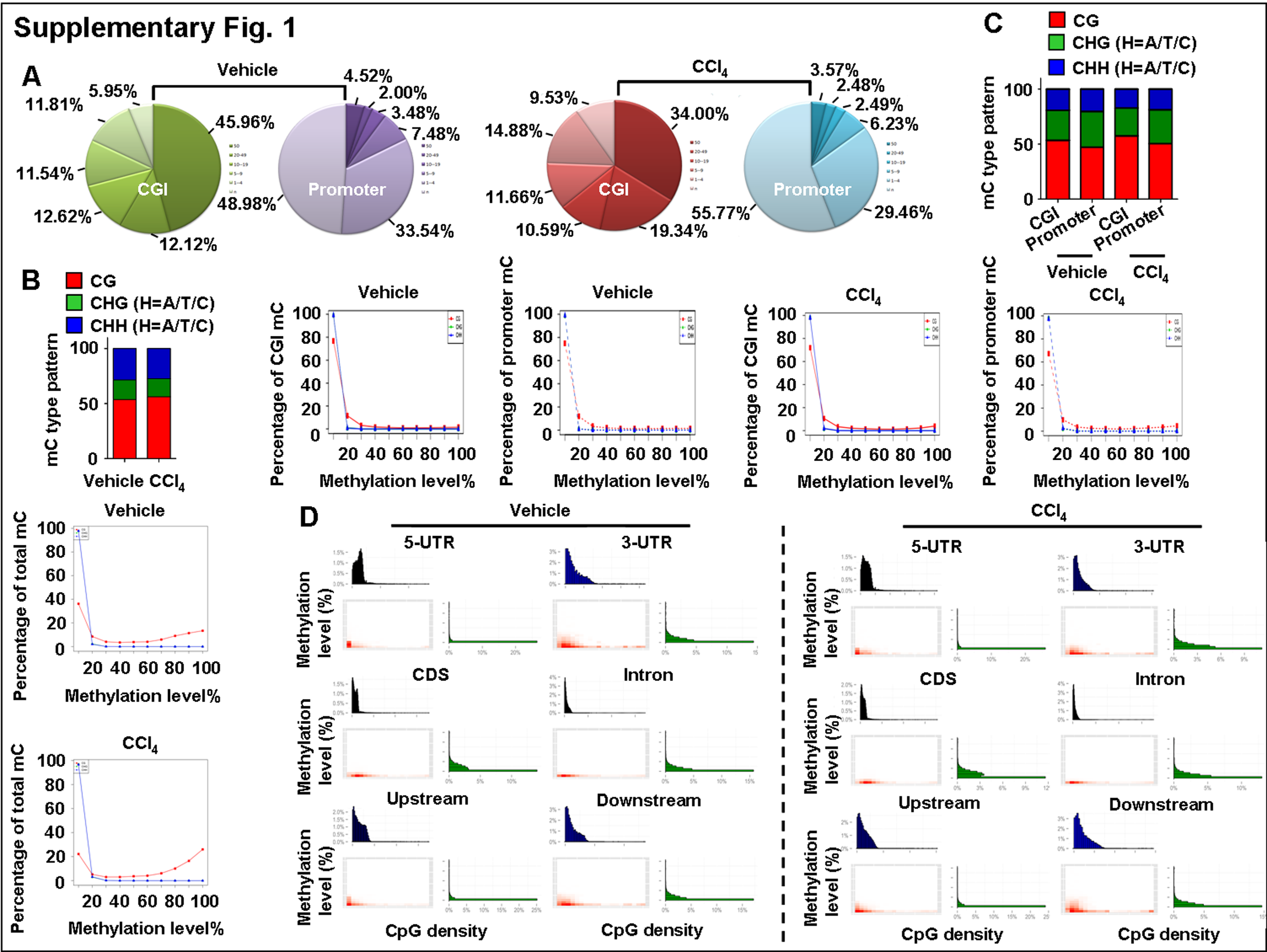

Supplement: Supplementary file 1 — Figure legends [file 41419_2018_1032_MOESM1_ESM.tif]

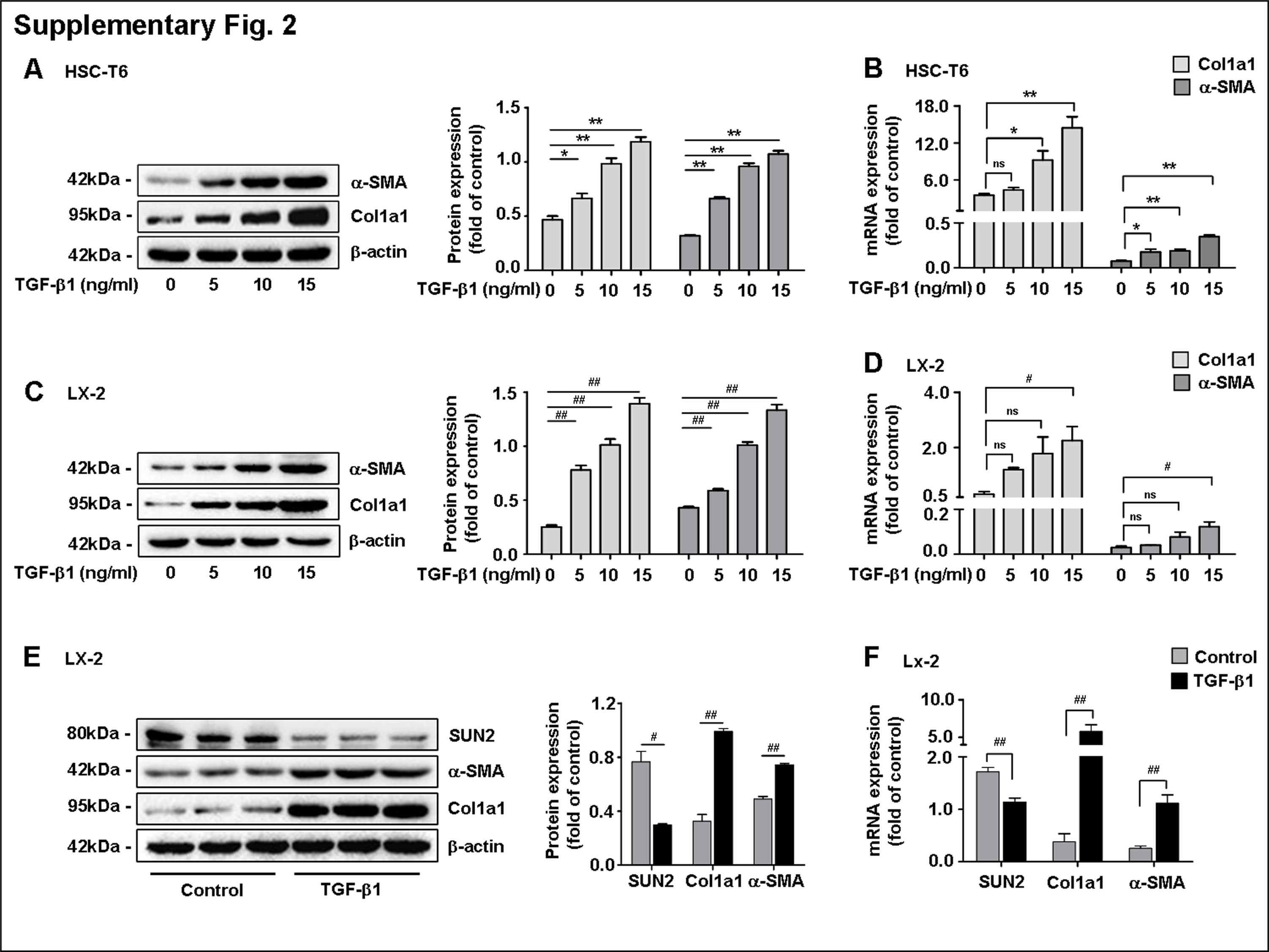

Supplement: Supplementary file 2 — Figure legends [file 41419_2018_1032_MOESM2_ESM.tif]

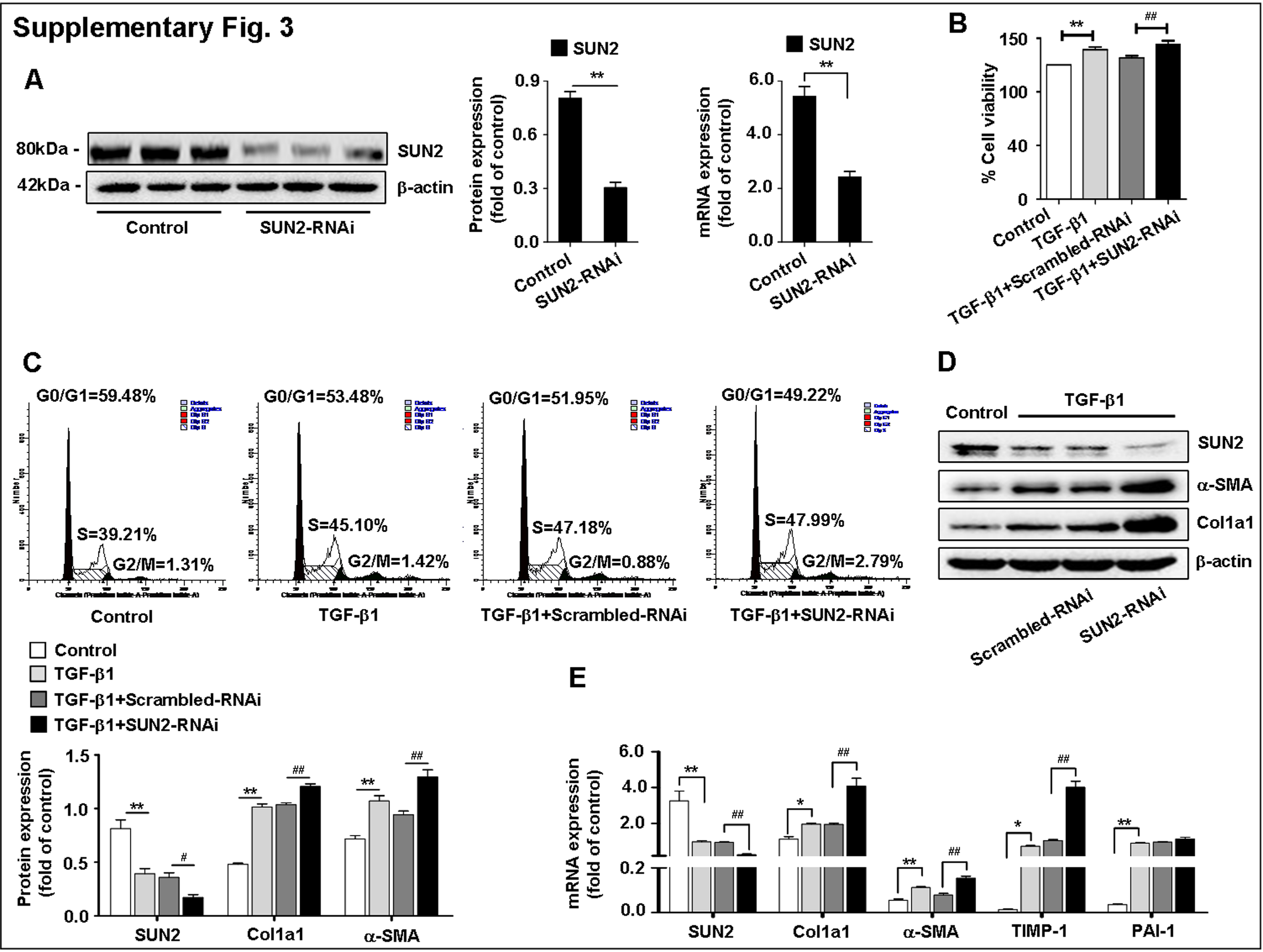

Supplement: Supplementary file 3 — Supplementary Figure 1 [file 41419_2018_1032_MOESM3_ESM.tif]

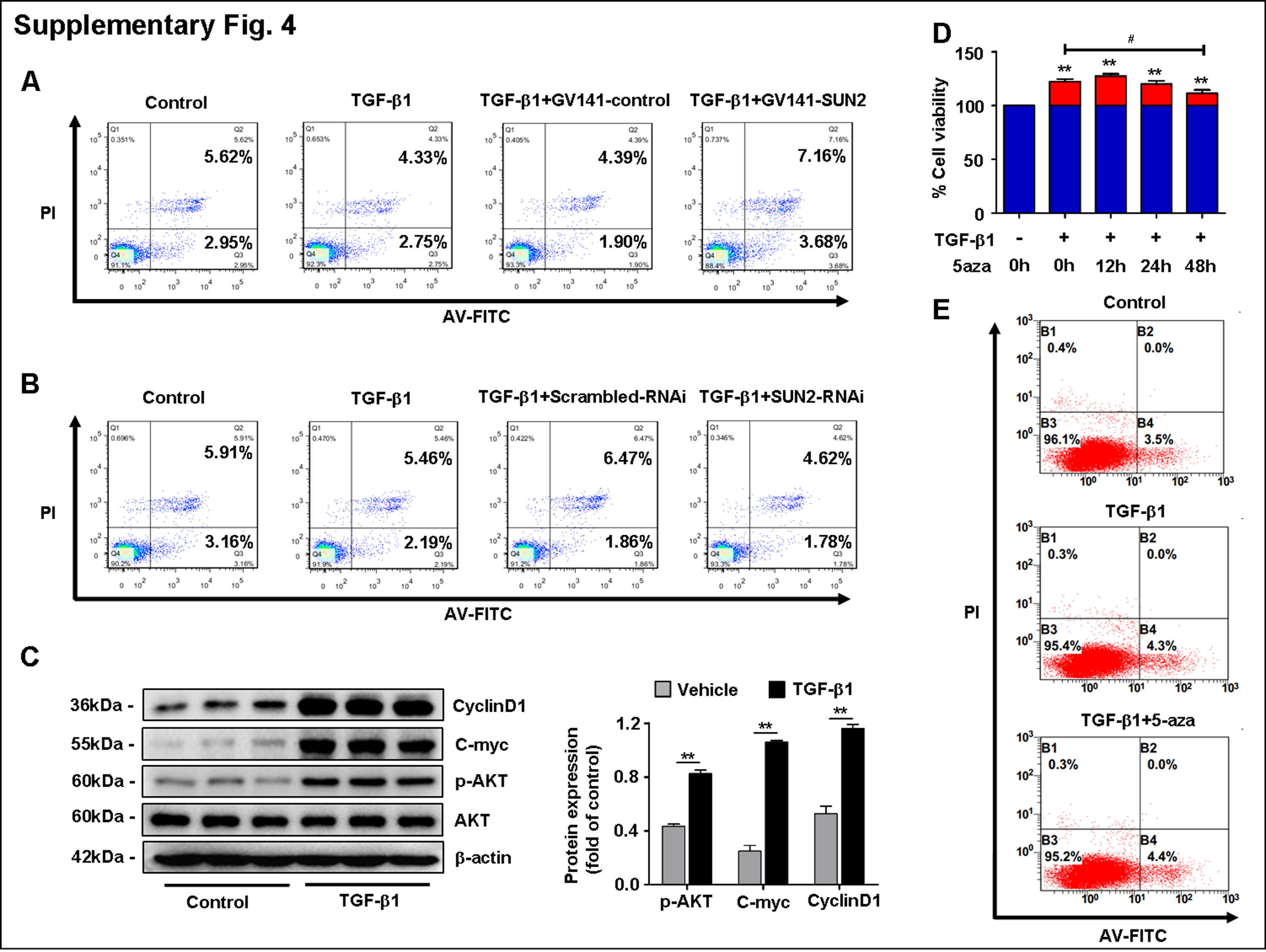

Supplement: Supplementary file 4 — Supplementary Figure 2 [file 41419_2018_1032_MOESM4_ESM.tif]

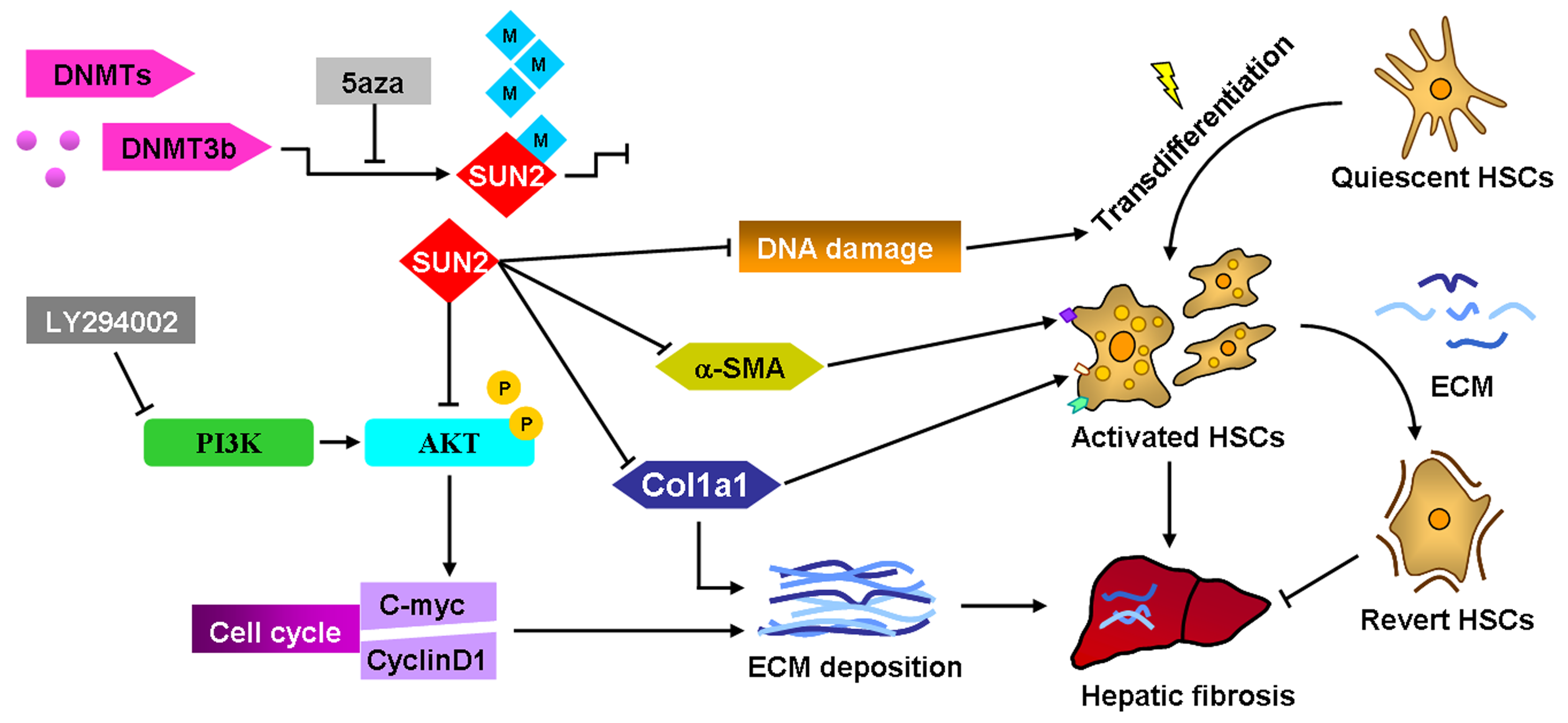

Supplement: Supplementary file 10 — Supplementary Table 2 [file 41419_2018_1032_MOESM10_ESM.tif]
