# Supplementary material for: Suppression of SUN2 by DNA methylation is associated with HSCs activation and hepatic fibrosis
Source: Cell Death Dis. 2018 Oct 3;9(10):1021. doi: 10.1038/s41419-018-1032-9 (PMC6170444; doi:10.1038/s41419-018-1032-9)
Supplement: Supplementary file 5 — Supplementary Figure 3 [file 41419_2018_1032_MOESM5_ESM.doc]

**Supplementary Figure legends**

**Supplementary Figure. 1. RRBS sequencing analysis.** **(A)** Coverage and depth statistics of reads in CGI and promoter from vehicle and hepatic fibrosis mice. Comparison about methylation levels of methylated-cytosine and different types (CG/CHG/CHH) of methylated-cytosine in **(B)** genome-wide, **(C)** CGI and promoter. **(D)** Methylation levels and CpG density patterns in diverse genomic regions showed in heatmaps. Red color gradient regions showed specific methylation level and the number of CpG in specific CpG density. Distribution of methylation levels projected onto Y-axis of heatmaps exhibited in bar charts in green color; Distribution of CpG density projected onto X-axis of heatmaps exhibited in bar charts in blue color.

**Supplementary Figure. 2. (A, B)** A dose-dependent response of α-SMA and Col1α1 mRNA and protein expression after TGF-β1 (5, 10, 15 ng/ml) stimulation in HSC-T6 cells by quantitative real-time PCR and western blot analysis. **(C, D)** A dose-dependent response of α-SMA and Col1α1 mRNA and protein expression after TGF-β1 (5, 10 and 15 ng/ml) stimulation in LX-2 cells by quantitative real-time PCR and western blot analysis. **(E, F)** Effects of TGF-β1 (15 ng/ml) on expression of SUN2, α-SMA and Col1α1 in LX-2 cells. Graphs show quantification of western blotting for SUN2, α-SMA and Col1α1. Bars represent mean ± SEM for 3 independent experiments *in vitro*. **p*<0.05, ***p*<0.01 versus Control in HSC-T6 cells; ^#^*p*<0.05, ^##^*p*<0.01 versus Control in LX-2 cells.

**Supplementary Figure. 3. Effects of SUN2 knockdown on activation and proliferation of HSC-T6 cells. (A)** Characterizations of SUN2 knockdown in HSC-T6 cells with SUN2-RNAi transfection. **(B)** Cell viability promoted in HSC-T6 cells transfected with SUN2-RNAi. **(C)** Flow cytometry showed cell numbers of S and G2 phases increased in TGF-β1-treated HSC-T6 with SUN2-RNAi transfection compared to Scrambled-RNAi *in vitro*. **(D, E)** knockdown SUN2 elevated the mRNA levels and protein expression of α-SMA, Col1α1, TGF-β1 and TIMP-1 in TGF-β1-activated HSC-T6 cells. Bars represent mean ± SEM for 3 independent experiments *in vitro*. **p*<0.05, ***p*<0.01 versus Control; ^#^*p*<0.05, ^##^*p*<0.01 versus TGF-β1+Scrambled-RNAi.

**Supplementary Figure. 4.** Flow cytometry revealed that enforcing or blocking expression of SUN2 exhibited no significantly effects on apoptosis of activated HSC-T6 cells *in vitro*. **(B)** Western blotting showed the levels of p-AKT, C-myc and CyclinD1 substantially elevated in activated HSC-T6 cells compared to Control. Graphs show quantification of western blotting for SUN2, p-AKT, C-myc and CyclinD1. Bars represent mean ± SEM for 3 independent experiments *in vitro*. **p*<0.05, ***p*<0.01 versus Control.
