# Supplementary material for: Suppression of SUN2 by DNA methylation is associated with HSCs activation and hepatic fibrosis
Source: Cell Death Dis. 2018 Oct 3;9(10):1021. doi: 10.1038/s41419-018-1032-9 (PMC6170444; doi:10.1038/s41419-018-1032-9)
Supplement: Supplementary file 6 — Supplementary Figure 4 [file 41419_2018_1032_MOESM6_ESM.doc]

**Supplementary Table 1. Identification of aberrantly methylated genes in DMRs.**

| **Chr** | **Start** | **End** | **Genes** | **DMR**  **start** | **DMR**  **end** | **Numbers of DMR** | **Methylation**  **level of M** | **Methylation**  **level of V** | **Fold of M/V** | **Corrected P-value** |
| --- | --- | --- | --- | --- | --- | --- | --- | --- | --- | --- |
| chr1 | 34114022 | 34116117 | Dst | 34114493 | 34114512 | 7 | 0.127 | 0.014 | 9.202 | 1.440E-06 |
| chr2 | 29802679 | 29802960 | Slc27a4 | 29802637 | 29802715 | 57 | 0.003 | 0.265 | 0.010 | 3.800E-152 |
| chr2 | 165504048 | 165514425 | Slc2a10 | 165510168 | 165510484 | 26 | 0.120 | 0.007 | 16.560 | 2.690E-24 |
| chr2 | 126593960 | 126594686 | Hdc | 126594472 | 126594645 | 67 | 0.039 | 0.188 | 0.206 | 1.810E-29 |
| chr2 | 170488040 | 170490114 | Cyp24a1 | 170488052 | 170488117 | 22 | 0.204 | 0.006 | 33.273 | 5.390E-30 |
| chr3 | 153206725 | 153411461 | St6galnac3 | 153351688 | 153351916 | 14 | 0.108 | 0.004 | 26.393 | 3.290E-12 |
| chr4 | 106915729 | 107009678 | Ssbp3 | 107001684 | 107001859 | 13 | 0.000 | 0.104 | 0.000 | 5.360E-05 |
| chr4 | 156170314 | 156170578 | Agrn | 156170408 | 156170469 | 5 | 0.158 | 0.000 | - | 4.110E-04 |
| chr5 | 35581059 | 35583059 | Acox3 | 35582881 | 35582908 | 16 | 0.010 | 0.128 | 0.080 | 7.710E-13 |
| chr5 | 35581226 | 35583574 | Rik | 35582881 | 35582908 | 16 | 0.010 | 0.128 | 0.080 | 7.710E-13 |
| chr5 | 115506868 | 115544569 | Pxn | 115544303 | 115544412 | 12 | 0.018 | 0.317 | 0.057 | 1.030E-21 |
| chr5 | 137300413 | 137302125 | Srrt | 137301530 | 137301556 | 13 | 0.433 | 0.036 | 11.988 | 1.380E-27 |
| chr6 | 47681058 | 47683058 | Rn4.5s | 47681978 | 47682856 | 55 | 0.160 | 0.041 | 3.882 | 1.760E-33 |
| chr6 | 86096685 | 86098575 | Add2 | 86096741 | 86096870 | 71 | 0.174 | 0.017 | 10.217 | 6.740E-68 |
| chr7 | 102555919 | 102557919 | Trim21 | 102557285 | 102557340 | 16 | 0.049 | 0.282 | 0.173 | 2.260E-23 |
| chr7 | 144049979 | 144443407 | Mir3470b | 144354100 | 144354172 | 24 | 0.167 | 0.012 | 14.222 | 2.020E-25 |
| chr7 | 144286766 | 144395462 | Shank2 | 144354100 | 144354172 | 24 | 0.167 | 0.012 | 14.222 | 2.020E-25 |
| chr8 | 108933531 | 108945101 | Zfhx3 | 108933597 | 108938596 | 16 | 0.140 | 0.021 | 6.760 | 2.420E-16 |
| chr8 | 108934859 | 108938938 | Mir3108 | 108933597 | 108938596 | 16 | 0.140 | 0.021 | 6.760 | 2.420E-16 |
| chr9 | 15301513 | 15306213 | Rik | 15306165 | 15306193 | 6 | 0.000 | 0.141 | 0.000 | 1.904E-04 |
| chr9 | 15304213 | 15306213 | Taf1d | 15306165 | 15306193 | 6 | 0.000 | 0.141 | 0.000 | 1.904E-04 |
| chr9 | 15306036 | 15308167 | Rik | 15306165 | 15306193 | 6 | 0.000 | 0.141 | 0.000 | 1.904E-04 |
| chr11 | 69398234 | 69400234 | Tmem88 | 69399769 | 69399822 | 44 | 0.340 | 0.002 | 157.958 | 2.420E-118 |
| chr11 | 69398517 | 69399825 | Kdm6b | 69399769 | 69399822 | 44 | 0.340 | 0.002 | 157.958 | 2.420E-118 |
| chr11 | 103774348 | 103808149 | Wnt3 | 103800266 | 103800339 | 13 | 0.132 | 0.003 | 46.108 | 4.770E-17 |
| chr11 | 115786175 | 115788765 | Rik | 115786287 | 115788755 | 30 | 0.184 | 0.032 | 5.688 | 1.400E-25 |
| chr11 | 117359470 | 117360422 | Sept9 | 117359502 | 117359601 | 10 | 0.187 | 0.009 | 20.702 | 6.330E-27 |
| chr11 | 118073515 | 118073888 | Dnah17 | 118073685 | 118073742 | 35 | 0.227 | 0.041 | 5.549 | 2.220E-26 |
| chr14 | 54408502 | 54409004 | Slc7a7 | 54408529 | 54408842 | 110 | 0.048 | 0.160 | 0.299 | 1.990E-61 |
| chr15 | 36174081 | 36174986 | Polr2k | 36174167 | 36174237 | 27 | 0.004 | 0.122 | 0.030 | 1.050E-22 |
| chr15 | 37397453 | 37563390 | Ncald | 37515593 | 37515629 | 8 | 0.125 | 0.009 | 13.825 | 1.090E-06 |
| chr15 | 76703436 | 76704239 | Mfsd3 | 76703896 | 76704034 | 78 | 0.116 | 0.011 | 10.993 | 4.060E-50 |
| chr15 | 76703865 | 76704062 | Recql4 | 76703896 | 76704034 | 78 | 0.116 | 0.011 | 10.993 | 4.060E-50 |
| chr15 | 76708739 | 76710739 | Lrrc14 | 76710470 | 76710529 | 17 | 0.004 | 0.144 | 0.027 | 2.570E-17 |
| chr15 | 76710392 | 76710559 | Recql4 | 76710470 | 76710529 | 17 | 0.004 | 0.144 | 0.027 | 2.570E-17 |
| *chr15* | *79737159* | *79738519* | *SUN2* | *79737437* | *79737572* | *33* | *0.113* | *0.006* | *17.931* | *1.850E-17* |
| chr15 | 102217773 | 102217997 | Itgb7 | 102217918 | 102217937 | 6 | 0.000 | 0.309 | 0.000 | 2.020E-12 |
| chr17 | 24504019 | 24506020 | Caskin1 | 24505053 | 24505142 | 26 | 0.311 | 0.035 | 8.796 | 2.640E-56 |
| chrUn_JH_584304584304 | 50673 | 61689 | Pisd-ps3 | 48849 | 68641 | 1014 | 0.023 | 0.146 | 0.157 | 0.000E+00 |
| chrY | 90783441 | 90785441 | Erdr1 | 90784525 | 90784584 | 26 | 0.191 | 0.064 | 3.003 | 9.430E-09 |

(DMRs, differentially methylated regions; M, CCl_4_-induced hepatic fibrosis mice; U, vehicle mice)
