# Supplementary material for: Suppression of SUN2 by DNA methylation is associated with HSCs activation and hepatic fibrosis
Source: Cell Death Dis. 2018 Oct 3;9(10):1021. doi: 10.1038/s41419-018-1032-9 (PMC6170444; doi:10.1038/s41419-018-1032-9)
Supplement: Supplementary file 7 — Supplementary Figure legends [file 41419_2018_1032_MOESM7_ESM.doc]

**Supplementary Table 2. KEGG enrichment of hypermethylated genes.**

| **Term** | **Database** | **Id** |
| --- | --- | --- |
| Fatty acid degradation | KEGG PATHWAY | mmu00071 |
| Steroid biosynthesis | KEGG PATHWAY | mmu00100 |
| Histidine metabolism | KEGG PATHWAY | mmu00340 |
| alpha-Linolenic acid metabolism | KEGG PATHWAY | mmu00592 |
| Glycosphingolipid biosynthesis - ganglio series | KEGG PATHWAY | mmu00604 |
| Biosynthesis of unsaturated fatty acids | KEGG PATHWAY | mmu01040 |
| Metabolic pathways | KEGG PATHWAY | mmu01100 |
| Fatty acid metabolism | KEGG PATHWAY | mmu01212 |
| PPAR signaling pathway | KEGG PATHWAY | mmu03320 |
| cAMP signaling pathway | KEGG PATHWAY | mmu04024 |
| Chemokine signaling pathway | KEGG PATHWAY | mmu04062 |
| Peroxisome | KEGG PATHWAY | mmu04146 |
| *PI3K-Akt signaling pathway* | *KEGG PATHWAY* | *mmu04151* |
| Wnt signaling pathway | KEGG PATHWAY | mmu04310 |
| Hedgehog signaling pathway | KEGG PATHWAY | mmu04340 |
| VEGF signaling pathway | KEGG PATHWAY | mmu04370 |
| Hippo signaling pathway | KEGG PATHWAY | mmu04390 |
| Focal adhesion | KEGG PATHWAY | mmu04510 |
| ECM-receptor interaction | KEGG PATHWAY | mmu04512 |
| Cell adhesion molecules | KEGG PATHWAY | mmu04514 |
| Signaling pathways regulating  pluripotency of stem cells | KEGG PATHWAY | mmu04550 |
| Leukocyte transendothelial migration | KEGG PATHWAY | mmu04670 |
| Intestinal immune network for IgA production | KEGG PATHWAY | mmu04672 |
| Glutamatergic synapse | KEGG PATHWAY | mmu04724 |
| Regulation of actin cytoskeleton | KEGG PATHWAY | mmu04810 |
| Melanogenesis | KEGG PATHWAY | mmu04916 |
| Protein digestion and absorption | KEGG PATHWAY | mmu04974 |
| Fat digestion and absorption | KEGG PATHWAY | mmu04975 |
| Huntington's disease | KEGG PATHWAY | mmu05016 |
| Bacterial invasion of epithelial cells | KEGG PATHWAY | mmu05100 |
| HTLV-I infection | KEGG PATHWAY | mmu05166 |
| Pathways in cancer | KEGG PATHWAY | mmu05200 |
| Transcriptional misregulation in cancer | KEGG PATHWAY | mmu05202 |
| Viral carcinogenesis | KEGG PATHWAY | mmu05203 |
| Proteoglycans in cancer | KEGG PATHWAY | mmu05205 |
| MicroRNAs in cancer | KEGG PATHWAY | mmu05206 |
| Basal cell carcinoma | KEGG PATHWAY | mmu05217 |
| Systemic lupus erythematosus | KEGG PATHWAY | mmu05322 |
| Hypertrophic cardiomyopathy | KEGG PATHWAY | mmu05410 |
| Arrhythmogenic right ventricular cardiomyopathy | KEGG PATHWAY | mmu05412 |
| Dilated cardiomyopathy | KEGG PATHWAY | mmu05414 |
