# Supplementary material for: Suppression of SUN2 by DNA methylation is associated with HSCs activation and hepatic fibrosis
Source: Cell Death Dis. 2018 Oct 3;9(10):1021. doi: 10.1038/s41419-018-1032-9 (PMC6170444; doi:10.1038/s41419-018-1032-9)
Supplement: Supplementary file 8 — Supplementary Figure legends [file 41419_2018_1032_MOESM8_ESM.doc]

**Supplementary Table 3A. Primers for quantitative real-time PCR.**

| **Gene** | **Forward** | **Reverse** |
| --- | --- | --- |
| **Mouse** | | |
| SUN2 | 5’-TGTGGTTCCTTCTGCTCTTG-3’ | 5’-GTCTCTGGATTCCCACACCT-3’ |
| Collα1 | 5’-TGTAAACTCCCTCCACCCCA-3' | 5’-TCGTCTGTTTCCAGGGTTGG-3’ |
| α-SMA | 5’-CGGGCTTTGCTGGTGATG-3’ | 5’-CCCTCGATGGATGGGAAA-3’ |
| TGF-β1 | 5’-CTCCCGTGGCTTCTAGTGC-3’ | 5’-GCCTTAGTTTGGACAGGATCTG-3’ |
| TIMP-1 | 5’-GCAACTCGGACCTGGTCATAA-3’ | 5’-CGGCCCGTGATGAGAAACT-3’ |
| PAI-1 | 5’-TCCAGAAGCAGAGAGGGAAA-3’ | 5’-CAGTGCAGCTCTGACTCACC-3’ |
| GAPDH | 5’-GGACCTCATGGCCTACATGG-3’ | 5’-TAGGGCCTCTCTTGCTCAGT-3’ |
| **Rat** | | |
| SUN2 | 5’-TTCTCTGGGACAGACACTGC-3’ | 5’-ATCCACCATTCCAATACGGT-3’ |
| Collα1 | 5’-GATCCTGCCGATGTCGCTAT-3’ | 5’-TGTAGGCTACGCTGTTCTTGCA-3’ |
| α-SMA | 5’-CGAAGCGCAGAGCAAGAGA-3’ | 5’-CATGTCGTCCCAGTTGGTGAT-3’ |
| TIMP-1 | 5’-TCCCCAGAAATCATCGAGAC-3’ | 5’-TCAGATTATGCCAGGGAACC-3’ |
| PAI-1 | 5’-CAGCGCCTGTTCCACAAGTC-3’ | 5’-TGTCGTACTCGTGCCCATCC-3’ |
| GAPDH | 5’-ACCACAGTCCATGCCATCAC-3’ | 5’-TCCACCACCCTGTTGCTGTA-3’ |
| **Human** | | |
| SUN2 | 5’-TGCCCTGAAGGAGGATTTC-3’ | 5’-TCATGCTTTGCCACTCTGAC-3’ |
| Col1α1 | 5’-CCCGGGTTTCAGAGACAACTTC-3’ | 5’-TCCACATGCTTTATTCCAGCAATC-3’ |
| α-SMA | 5’-AGGCACCCCTGAACCCCAA-3’ | 5’-CAGCACCGCCTGGATAGCC-3’ |
| β-actin | 5’-GCCAACACAGTGCTGTCTGG-3’ | 5’-CTCAGGAGGAGCAATGATCTTG-3’ |

**Supplementary Table 3B. M and U primers of SUN2 for MSP.**

| **Primer** | **Forward** | **Reverse** |
| --- | --- | --- |
| SUN2-M | 5’-GAATTTCGATACGGGAATAGTTAC-3’ | 5’-GCAAAAAAATTAAAATAACCCGAC-3’ |
| SUN2-U | 5’-AATTTTGATATGGGAATAGTTATGG-3’ | 5’-ACAAAAAAATTAAAATAACCCAAC-3’ |

(M, Methylated; U, Unmethylated)
