# Supplementary material for: Suppression of SUN2 by DNA methylation is associated with HSCs activation and hepatic fibrosis
Source: Cell Death Dis. 2018 Oct 3;9(10):1021. doi: 10.1038/s41419-018-1032-9 (PMC6170444; doi:10.1038/s41419-018-1032-9)
Supplement: Supplementary file 9 — Supplementary Table 1 [file 41419_2018_1032_MOESM9_ESM.doc]

**Supplementary File 1. RRBS sequencing experimental procedures and bioinformatics analysis.**

**(A) Procedures**


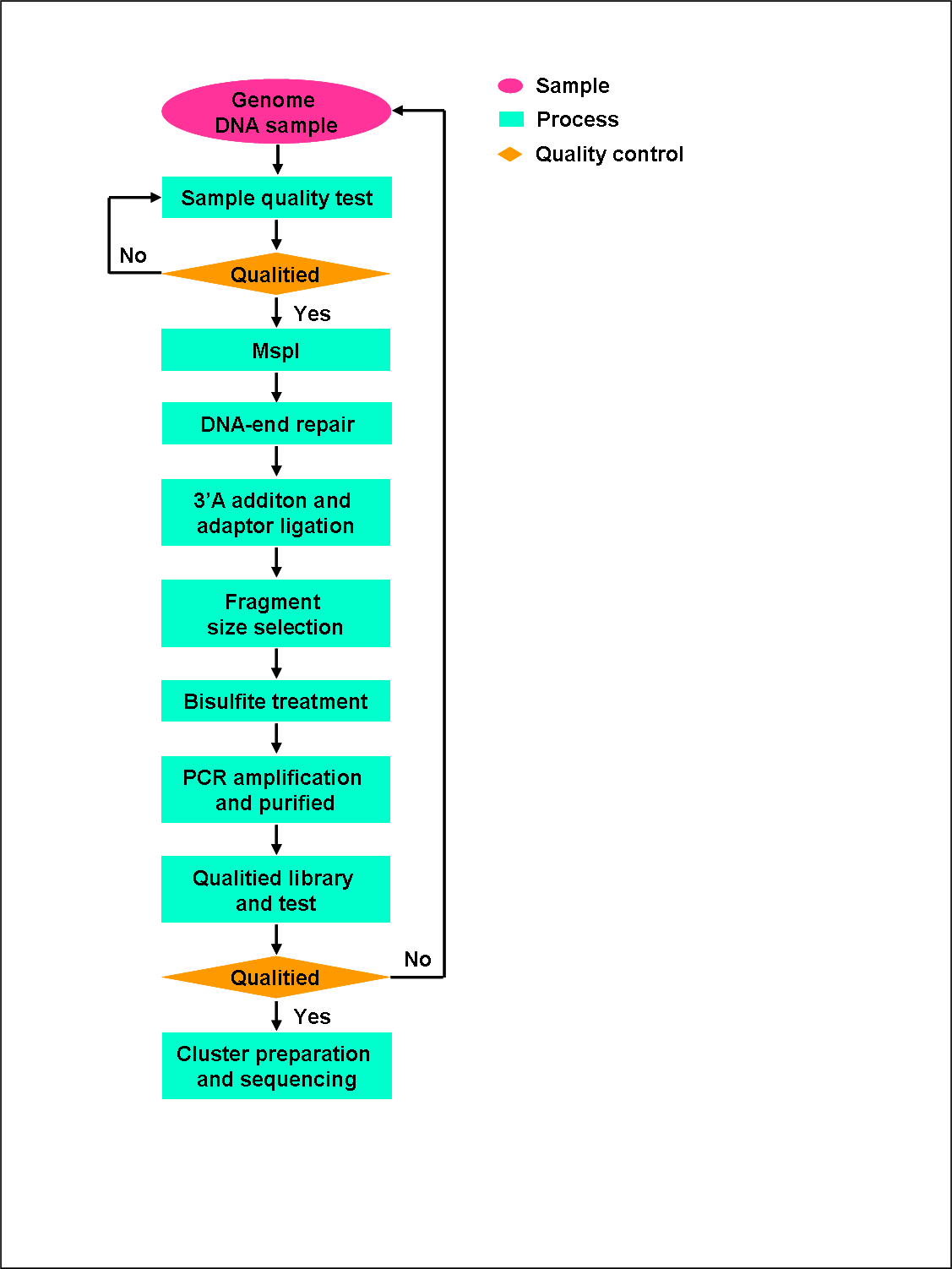


**(B) Bioinformatics analysis**


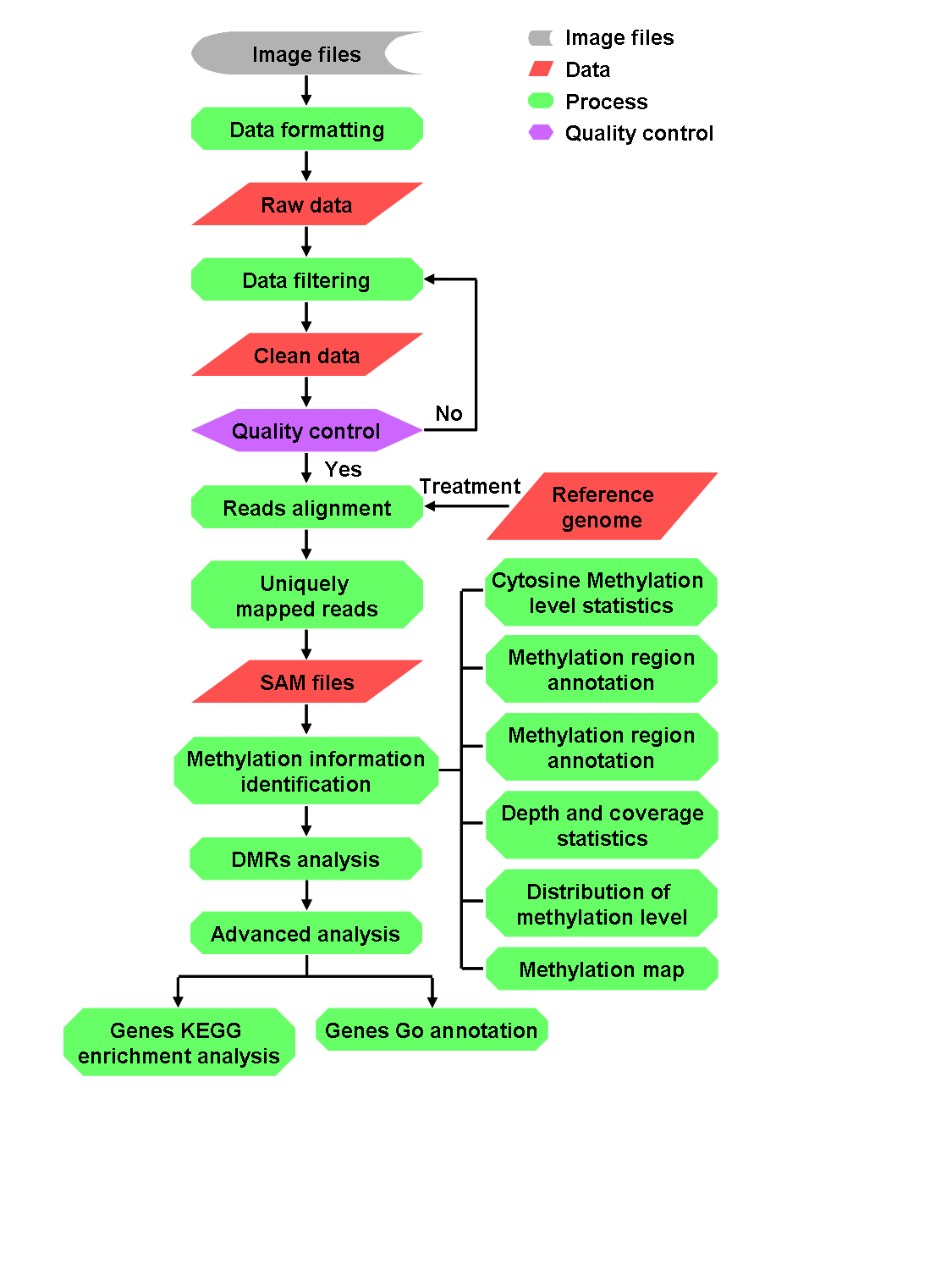


(SAM, sequence alignment map; KEGG, kyoto encyclopedia of genes and genomes)
